# The association between the restriction of daily life and depression during the COVID-19 pandemic in Korea: a nationwide based survey

**DOI:** 10.1038/s41598-022-21301-5

**Published:** 2022-10-21

**Authors:** Sunwoo Cho, Hyo Rim Ju, Hyoungseok Oh, Eun-Suk Choi, Jung Ah Lee

**Affiliations:** 1 Workplace Health Institute, Total Health Care Center, Kangbuk Samsung Hospital, Sungkyunkwan University School of Medicine, B1, 55 Sejong-daero, Jung-gu, Seoul, South Korea; 2 Department of Epidemiology and Biostatistics, Arnold School of Public Health, University of South Carolina, Columbia, SC USA; 3 Department of Family Medicine, Dankook University Hospital, Dankook University College of Medicine, Cheonan, South Korea

**Keywords:** Depression, Risk factors

## Abstract

The coronavirus (COVID-19) pandemic has led to substantial daily life changes for people worldwide. We investigated the association between daily life restrictions and depression during the COVID-19 pandemic based on the Korea Community Health Survey. Daily life restrictions were evaluated using a questionnaire to population into three restriction categories: no/slightly, moderately, and severely. Depression was assessed by the Korean version of the Patient Health Questionnaire-9 (PHQ-9). Chi-square tests and Fisher’s exact tests were used to compare the demographic characteristics of individuals with and without depression. Logistic regression was used to assess the association between the severity of daily life restrictions and the prevalence of depression. The prevalence of depression was 2.4% in the total population: 5.7% in the severely restricted group and 2.7% in the moderately restricted group. After adjusting for age, sex, educational level, income, marital status, and employment status, the severely restricted group was more likely to have depression than was the no change/slightly restricted group (OR = 2.40, 95% CI 2.16–2.67, p < 0.001). Employers with severely restricted daily life exhibited a higher OR for depression compared to the no/slightly restricted group (OR = 3.24, 95% CI 2.37–4.45, p < 0.001). It is necessary to consider the mental health of vulnerable affected by the COVID-19 pandemic.

## Introduction

Coronavirus disease 2019 (COVID-19) began its spread in November 2019 and remains a worldwide concern to date^1^. Before the Omicron variants spread widely, the government of each nation expended an effort to reduce the spread of COVID-19 by imposing various policy interventions. To control COVID-19, the Korea Disease Control and Prevention Agency implemented the mandatory use of face masks in public places, a social distancing policy, extensive COVID-19 screening tests, COVID-19 contact tracing, and self-quarantine^2^. The social distancing policy was first implemented in Korea on March 21, 2020. It could be adjusted every two weeks according to the number of persons infected and deaths from COVID-19^3^. The social distancing level is comprised of four or five stages, whereby higher levels impose greater restrictions due to aggravated COVID-19 spread. These restrictions include limitations on the number of people in a gathering, permitted crowd numbers at sports venues, electronic log systems requirements, business hour restrictions, and prohibitions on eating food in indoor public places^4^, which could affect the social activity of people^5^.

Previous studies reported the association between COVID-19 and mental illness including mental disorders such as depressive episodes, anxiety disorders, and insomnia^6–10^. A meta-analysis in one country reported the pooled prevalence of depression was 47% and was associated with age, sex, lifestyle factors, and socioeconomic status^8^. One study reported depressed people were younger, lower educated, and married than non-depressed people in the quarantined population^11^. Because the prevalence of depression has been noticeably related to lifestyle factors (physical activity, nutrition, sleep, and stress management)^12^, it would be important to address those factors on depression, especially during the pandemic situation.

A recent study demonstrated mediating effects of daily life change and anxiety related to depression based on occupational types during the COVID-19 pandemic in South Korea^10^. In particular, compared to Western countries, South Korea has implemented a social distancing policy with stratified stages for a long time. Thus, it is possible that continuous social distancing has had an impact on mental health such as depression. Also, since depression could be associated with lifestyle factors such as physical activity, dietary habits, and alcohol consumption^13–15^, daily life changes due to COVID-19 might affect the mental health of the general population. Therefore, we evaluated the relationship between daily life restrictions and daily life changes, and depression using the Korea Community Health Survey (KCHS). Furthermore, we investigated the association between daily life restrictions due to COVID-19 and depression according to employment status.

## Methods

### Data design and setting of the study

**Figure 1:**
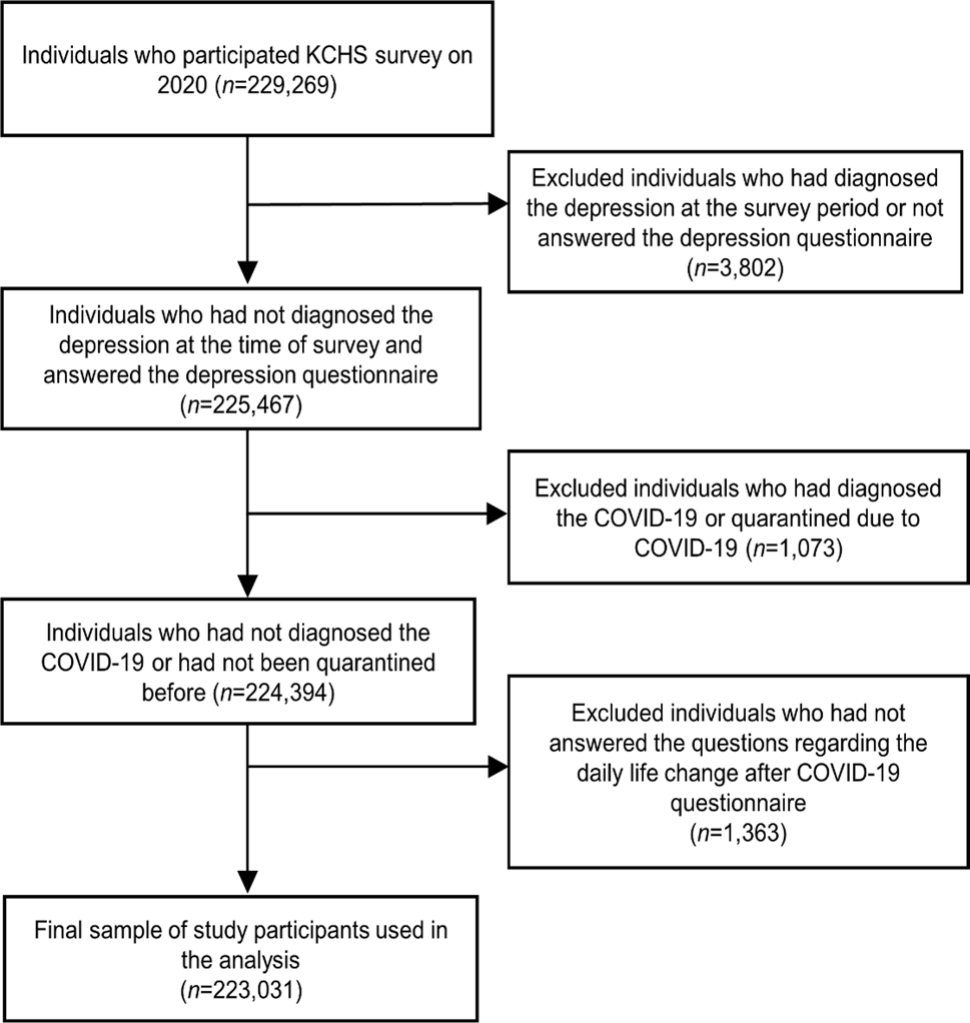
Flow chart of the study population.

The KCHS is a nationwide, community-based survey conducted since 2008 to investigate public health status and health behaviors of individuals aged 19 years or older in provinces of 254 districts of 17 metropolitan cities. It was designed by the Korea Centers for Disease Control and Prevention (KCDC) and assisted by the Ministry of Health and Welfare of South Korea. Systematic sampling has been employed to sample households and those are selected based on local districts. To achieve the same confidence level and the degree of accuracy of population, approximately 900 individuals per district were selected by a multistage probability sampling design based on community classification and type of residence^16^. In 2020, a total of 229,269 participants in the survey from August 16 to October 31. We excluded 6238 participants who were diagnosed with COVID-19 or quarantined due to COVID-19 (n = 1073), previously diagnosed with depressive disorder or with missing data on the PHQ-9 questionnaire (n = 3802), or with missing data on change in daily life during the COVID-19 pandemic on the questionnaire (n = 1363). Finally, a total of 223,031 participants were included in the analysis (Fig. 1). This study was approved by the Institutional Review Board of Kangbuk Samsung Hospital, which waived the requirement for informed consent due to the use of anonymized KCHS data open to public (IRB No. 2022-03-062).

The demographic characteristics of the study population were investigated, namely age, sex, educational level, income, marital status, and employment status. Educational level was categorized as obtaining less than junior high school graduation (< 9 years), having graduated high school (9 years), and a junior higher educational level than junior high school graduate (> 9 years). Income was categorized as follows: < 2,000,000 KRW (1 USD = 1233 KRW), 2,000,000–3,999,999 KRW, and ≥ 4,000,000 KRW. Marital status was categorized as married/cohabiting, separated/widowed/divorced, and never-married single. Employment status was categorized as employer, employee, and unemployed (including full-time students and housewives).

Health behaviors were investigated using established questionnaires. Physical activity was calculated as metabolic equivalent (MET) minutes per week according to the International Physical Activity Questionnaire. We classified physical activity as low (< 600 MET minutes per week), moderate (600–2999 MET min per week), and high physical activity (≥ 3000 MET min per week). Smoking status was categorized as non-smokers, ex-smokers, and current smokers. Non-smokers were defined individuals who had never smoked or had smoked fewer than 100 cigarettes in their lifetime. Ex-smokers were defined individuals who had a lifetime history of smoking 100 or more cigarettes but did not currently smoke. Current smokers were defined individuals who had a lifetime history of smoking 100 or more cigarettes and currently smoked. Risky alcohol consumption was defined as two or more weekly episodes of consuming more than 7 standard drinks for men and 5 standard drinks for women.

### Assessment of daily life change during the COVID-19 pandemic and depression

Daily life changes were assessed by the following question: “Assume that daily life before the COVID-19 outbreak is 100 points. What score would you give current daily life compared to before the COVID-19 outbreak? Zero points indicates complete restriction of daily life. The smaller the score, the greater the change in your daily life restrictions. The perfect score would be 100, if you do not feel any additional restriction at the current moment during the COVID-19 pandemic.” We divided these response scores into three categories: no change or slightly restricted (70–100), moderately restricted (30–60), and severely restricted (0–20). Additionally, specific daily life changes were examined, including physical activity, fast food consumption, delivery food, alcohol consumption, and meeting neighbors. For each specific category, individuals could answer whether their current amount of time spent on each specific activity increased, decreased, or remained the same compared to before COVID-19 pandemic.

The Korean version of the Patient Health Questionnaire-9 (PHQ-9) was used to determine the possible presence of depression among participants^17^. The PHQ-9 is a self-reported questionnaire for screening depressive disorders. It addresses liitle interest or pleasure in doing things, depressed or hopeless, suicidal ideation, or harming others for the previous two weeks. The respondent rates each item from 0 (not to all) to 3 (nearly every day). The maximum possible score is 27 (9 questions × 3 points maximum denoting the most severe symptom). The final score range can be divided into four levels: 0–4 (none), 5–9 (mild), 10–14 (moderate), 15–19 (moderately severe), and 20–27 (severe). The presence of depression based on the Korean PHQ-9 was defined as a score of 10 or more, which is highly consistent with the DSM-IV diagnoses^18^.

### Statistical analysis

The statistical analysis considered the complex sample design to obtain accurate results and interpretations. Basic characteristics of the study population are presented as unweighted samples of individuals and weighted percentages. We present the prevalence of depression as unweighted numbers with weighted percentages. We used the chi-square test or Fisher’s exact test to compare the two groups according to whether they had depression.

Logistic regression analyses were conducted to calculate the odds ratios (ORs) and 95% confidence intervals (CIs) of the prevalence of depression according to the total daily life change score and five categories of specific daily life changes. Model 1 was a univariate analysis to evaluate the association between depression and daily life restrictions. Model 2 adjusted for age and sex. Model 3 additionally adjusted for covariates of age, sex, marital status, employment status, income, and educational status. We also calculated the marginal effect of daily life changes on the prevalence of depression, which could clarify the effect of each factor associated with depression in model 3. Marginal effects show the change in probability when the predictor or independent variable increases by one unit^19,20^. Since covariates related to depression were all binary variables, the change was from the base category to the targeted category as one unit. All statistical analyses were performed using STATA 16.1 (Stata Corporation, College Station, Texas, USA). Statistical significance was established at a two-tailed P < 0.05 level. All methods were performed in accordance with the relevant guidelines and regulations.

## Results

### Basic characteristics of study population

The basic characteristics of the study population are presented in Table 1. The average age was 48.8 years. Of the total population, 49.8% were male and 50.2% were female. The proportion of females was higher among individuals with depression (36.2% in males and 63.8% in females). A higher proportion of individuals with depression reported a low educational level (< 9 years of education, 20.1% without depression and 32.3% with depression), low income level (17.8% without depression and 34.6% with depression), and unemployed status (37.9% without depression and 54.4% with depression).

**Table 1:** Basic characteristics of study participants.

Variables	Total	No	Yes	*P* value^a^
		(N = 217,792)	(N = 5210)	
		Mean ± SE^b^	Mean ± SE	
Age (year)	48.8 ± 0.05	48.7 ± 0.05	49.9 ± 0.26	
BMI (kg/m^2^), self-reported	23.6 ± 0.01	23.6 ± 0.01	23.5 ± 0.06	

		N (%)^c^	N (%)	
**Sex**				
Male	101,715 (49.8)	100,020 (50.2)	1695 (36.2)	< 0.001
Female	121,287 (50.2)	117,772 (49.8)	3515 (63.8)	
**Education**				
> 9 years	84,172 (50.8)	82,719 (51.1)	1453 (39.3)	< 0.001
9 years	62,565 (28.7)	61,281 (28.8)	1284 (28.4)	
< 9 years	76,015 (20.4)	73,543 (20.1)	2472 (32.3)	
Missing	250 (0.1)			
**Income**				
≥ 4,000,000 KRW ^d^	105,557 (54.7)	103,829 (56.8)	1728 (40.7)	< 0.001
2–3,999,999 KRW	52,963 (24.7)	51,886 (25.4)	1077 (24.7)	
< 2,000,000 KRW	59,083 (17.6)	56,787 (17.8)	2296 (34.6)	
Missing	5399 (3.0)			
**Marital status**				
Married, cohabited	140,422 (60.8)	138,017 (61.3)	2405 (45.1)	< 0.001
Separated, divorced, bereaved	43,417 (14.4)	41,658 (14.1)	1759 (26.5)	
Never-married	39,048 (24.7)	38,007 (24.6)	1041 (28.4)	
Missing	115 (0.1)			
**Employment status**				
Employer^e^	87,260 (46.3)	85,828 (47.5)	1432 (35.8)	< 0.001
Employee	40,145 (14.2)	39,562 (14.6)	583 (9.8)	
Unemployed	86,616 (37.7)	83,568 (37.9)	3048 (54.4)	
Missing	8981 (1.8)			
**Physical activity (MET-minutes/week)**				
0–599	94,737 (40.3)	91,734 (39.9)	3003 (53.7)	< 0.001
600–2999	68,189 (32.9)	67,022 (33.1)	1167 (25.0)	
≥ 3000	59,923 (26.8)	58,887 (27.0)	1036 (21.3)	
Missing	153 (0.1)			
**Smoking**				
Never smoker	149,401 (65.3)	145,916 (65.4)	3485 (63.0)	< 0.001
Former smoker	37,576 (16.7)	36,824 (16.8)	752 (14.2)	
Current smoker	36,001 (18.0)	35,028 (17.8)	973 (22.8)	
Missing	24 (0.0)			
**Risky alcohol consumption**				
No	203,075 (89.9)	198,377 (89.9)	4498 (87.5)	< 0.001
Yes	19,932 (10.1)	19,416 (10.1)	516 (12.5)	
Missing	24 (0.0)			

^a^P values are calculated based on the resulting statistics of chi-square test or Fisher’s exact test.

^b^Weighted mean with standard error.

^c^Unweighted numbers with weighted percentage.

^d^1 USD = 1233.33 KRW in June 1st, 2020.

^e^Self-employed people are included in the employer for the employment status.

*BMI* body mass index, *KRW* Korean won, *MET-minutes* metabolic equivalent of task-minutes, *SE* standard error.

### Depression and daily life changes during the COVID-19 pandemic

Table 2 shows the prevalence of depression according to the total score and specific categories of daily life changes during the COVID-19 pandemic. The prevalence of depression was 2.4% and increased among individuals with severe daily life restrictions, 5.7% in the severely restricted group, and 2.7% in the moderately restricted group, respectively.

**Table 2:** Daily life restriction during COVID-19 pandemic in study population.

	Total	N (%)^a^		P value^b^
		No	Yes	
Total		217,817 (97.6)	5214 (2.4)	
**Total score of daily life restriction during COVID-19 pandemic**				
70–100 (no change)	76,266 (34.34)	74,365 (97.51)	1901 (2.49)	< 0.001
30–60 (moderately restricted)	123,515 (56.62)	120,245 (97.35)	3270 (2.65)	
0–20 (severely restricted)	22,301 (10.04)	21,306 (94.33)	1265 (5.67)	
**Specific daily life change during COVID-19 pandemic**				
**Physical activity**				
Increased/Same	128,861 (57.7)	126,168 (97.91)	2693 (2.09)	< 0.001
Decreased	94,149 (42.3)	91,631 (97.33)	2518 (2.67)	
**Fast food consumption**				
Decreased/Same	196,368 (88.1)	192,401 (97.80)	4317 (2.20)	< 0.001
Increased	26,601 (11.9)	25,705 (96.63)	896 (3.37)	
**Delivery food**				
Decreased/Same	177,599 (79.6)	173,575(97.73)	4024 (2.27)	< 0.001
Increased	45,367 (20.4)	44,177 (97.38)	1190 (2.62)	
**Alcohol consumption**				
Decreased/Same	215,717 (96.75)	210,849 (97.74)	4868 (2.26)	< 0.001
Increased	7246 (3.25)	6900 (95.22)	346 (4.78)	
**Meeting neighbors**				
Increased/Same	39,952 (17.91)	38,554 (96.50)	1398 (3.50)	< 0.001
Decreased	183,063 (82.09)	179,247 (97.92)	3816 (2.08)	

^a^Unweighted numbers with weighted percentage.

^b^P values are calculated based on the resulting statistics of chi-square test or Fisher’s exact test.

Table 3 shows the individual odd ratios (ORs) and marginal effects for depression according to daily life changes. After adjusting for age, sex, educational level, income, marital status, and employment status, the severely restricted group was more than twice as likely to exhibit depression than the no change/slightly restricted group (OR = 2.40, 95% CI 2.16–2.67) in model 3. For the specific category of daily life changes, the OR for depression was increased among individuals with decreased physical activity (OR = 1.45, 95% CI 1.36–1.56), increased fast-food consumption (OR = 2.00, 95% CI 1.81–2.20), increased consumption of delivery food (OR = 1.62, 95% CI 1.47–1.78), and increased alcohol consumption (OR = 3.03, 95% CI 2.65–3.47). In contrast, the OR for depression was lower among individuals with decreased meetings with neighbors than among those with unchanged meeting behavior or who had increased their time spent with neighbors (OR = 0.66, 95% CI 0.60–0.71).

**Table 3:** Logistic regression analysis of depression during the COVID-19 pandemic.

	Model 1^a^		Model 2^b^		Model 3^c^		Marginal effect^d^ (CI, 95%)
	OR (CI, 95%)	P value	OR (CI, 95%)	*P* value^†^	OR (CI, 95%)	*P* value	
**Total score of daily life restriction during the COVID-19 pandemic**							
70–100 (no change)	1		1		1		1
30–60 (moderately restricted)	1.10 (1.01–1.19)	0.029	1.07 (0.99–1.17)	0.099	1.14 (1.04–1.24)	< 0.001	0.0023 (0.0008, 0.0037)
0–20 (severely restricted)	2.46 (2.22–2.73)	< 0.001	2.33 (2.10–2.58)	< 0.001	2.40 (2.16–2.67)	< 0.001	0.0228 (0.0194, 0.0262)
**Specific daily life change during the COVID-19 outbreak**							
**Physical activity**							
Increased/Same	1		1		1		1
Decreased	1.38 (1.28–1.47)	< 0.001	1.35 (1.26–1.45)	< 0.001	1.45 (1.36–1.56)	< 0.001	0.0074 (0.0060, 0.0088)
**Fast food consumption**							
Decreased/Same	1		1		1		1
Increased	1.62 (1.48–1.77)	< 0.001	1.77 (1.61–1.94)	< 0.001	2.00 (1.81–2.20)	< 0.001	0.0169 (0.0140, 0.0199)
**Delivery food**							
Decreased/Same	1		1		1		1
Increased	1.18 (1.09–1.28)	< 0.001	1.29 (1.18–1.40)	< 0.001	1.62 (1.47–1.78)	< 0.001	0.0104 (0.0081, 0.0127)
**Alcohol consumption**							
Decreased/Same	1		1		1		1
Increased	2.44 (2.14–2.77)	< 0.001	2.64 (2.32–3.01)	< 0.001	3.03 (2.65–3.47)	< 0.001	0.0363 (0.0296, 0.0430)
**Meeting neighbors**							
Increased/Same	1		1		1		1
Decreased	0.57 (0.53–0.62)	< 0.001	0.56 (0.52–0.61)	< 0.001	0.66 (0.60–0.71)	< 0.001	− 0.0097 (− 0.0119, − 0.0075)

^a^Model 1 is a univariate logistic regression analysis with the dependent variable as the occurrence of depression.

^b^Model 2 an adjusted logistic regression analysis for age and sex with the dependent variable as the occurrence of depression.

^c^Model 3 an adjusted logistic regression analysis for age, sex, marital status, employment status, income and educational status with the dependent variable as the occurrence of depression.

^d^The marginal effect is calculated based on the Model 3.

*OR* odds ratio, *CI* confidence interval.

To determine the clear individual effect of each factor that influenced the presence of depression, we focused on interpreting the marginal effects of model 3. Compared to the no/slightly restricted group, the moderately restricted group exhibited a 0.23% higher and the severely restricted group a 2.28% higher predicted probability of reporting depression. For specific daily life changes, the marginal effect of decreased physical activity, increased fast-food consumption, increased delivery food consumption, and increased alcohol consumption were associated with a 0.74%, 1.69%, 1.04%, and 3.63% increase in the predicted probability of depression, respectively. Otherwise, the predicted probability of depression decreased by 0.97% for individuals who reduced their time spent meeting neighbors.

### Depression and daily life changes according to employment status

Table 4 shows the level of restriction and depression according to employment status. Regardless of employment status, the ORs and predicted probabilities of depression increased in the severely restricted group compared with the no/slightly restricted group. Among the three categories of employment status, employers with severely restricted daily life exhibited triple the OR for depression compared to the no/slightly restricted group (OR = 3.24, 95% CI 2.37–4.45) and their marginal effect was an increase by 2.24%. Among unemployed individuals, both the moderately and severely restricted group exhibited increased ORs for depression versus the no/slightly restricted group (OR = 1.15, 95% CI 1.03–1.29 in moderately restricted group and OR = 2.26, 95% CI 1.96–2.60 in severely restricted group). The marginal effects were 0.37% in the moderately restricted group and 2.93% in the severely restricted group. Additionally, the Cochran-Armitage trend test showed that there were significant trend relationships between the severity of daily life restriction and depression for all three employment status.

**Table 4:** Logistic regression analysis^a^ of depression during the COVID19 pandemic according to employment status.

Total score of daily life change during COVID-19 pandemic	Prevalence of depression (%)	OR (CI, 95%)	P value	P for trend^b^	Marginal effect^c^
**Employee**					
70–100 (no change or slighted restricted)	2.17	1		< 0.001	1
30–60 (moderately restricted)	4.26	1.09 (0.94–1.26)	0.247		0.0012 (-0.0008, 0.0031)
0–20 (severely restricted)	1.33	2.24 (1.84–2.72)	< 0.001		0.0158 (0.0112, 0.0205)
**Employer**					
70–100 (no change or slighted restricted)	0.76	1		< 0.001	1
30–60 (moderately restricted)	1.54	1.19 (0.92–1.55)	0.198		0.0019 (-0.0009, 0.0048)
0–20 (severely restricted)	0.74	3.24 (2.37–4.45)	< 0.001		0.0224 (0.0152, 0.0295)
**Unemployed**					
70–100 (no change or slighted restricted)	5.43	1		< 0.001	1
30–60 (moderately restricted)	8.57	1.15 (1.03–1.29)	0.011		0.0037 (0.0009, 0.0062)
0–20 (severely restricted)	3.55	2.26 (1.96–2.60)	< 0.001		0.0293 (0.0233, 0.0352)

^a^An adjusted logistic regression analysis for age, sex, marital status, employment status, income and educational status with the dependent variable as the occurrence of depression according to employment status.

^b^The resulting P for trend is using the Cochran–Armitage test for trend.

^c^The marginal effect is calculated based on the adjusted logistic regression analysis in table.

## Discussion

In our study, the severe daily life restrictions during the COVID-19 pandemic were associated with increased depression in the Korean population. The total severity of daily life restrictions was associated with increased depression. In terms of specific daily life changes, the prevalence of depression was associated with reduced physical activity, increased consumption of fast food or delivery food, and increased alcohol consumption. Interestingly, reduced time spent meeting neighbors was correlated with decreased depression.

A recent study reported that the global prevalence of depressive disorders increased dramatically due to the COVID-19 pandemic^21^. The fear of COVID-19 infection and the social and economic effects of the COVID-19 pandemic could induce increased depression in populations. In Korea, the government implemented the mandatory use of face masks in public places, a social distancing policy, extensive COVID-19 screening tests, COVID-19 contact tracing, and self-quarantine. Since daily life restrictions occurred as a consequence of these policies, people who responded sensitively to government policies might have become more depressed.

Consistent with a previous study^10^, we found that people having a specific occupational status with highly affected by COVID-19 tended to have a higher prevalence of depression. In subgroup analysis, the OR for depression markedly increased in severely restricted employers than employees. Because the social distancing policy restricted operating hours and the number of people permitted to attend public and private facilities, employers running small private businesses may have been affected by those policies. In addition, the prevalence of depression was higher among the unemployed population (including students) in each group according to daily life restrictions; the marginal effect of depression was 2.9% among the severely restricted group. Unemployment status is a well-known cause of depression^22^ and affects on suicidal behaviors^23^. As a previous study reported the patterns of suicide during the COVID-19 pandemic^24^, assessing the risk of suicide attempts would be important among vulnerable populations.

For the specific category of daily life changes, decreased physical activity was associated with depression in our study. It is known that reduced physical activity can cause depression studies^25–27^ and sufficient physical activity has a protective effect against depression^28,29^. Also, our study showed that increased consumption of fast-food or delivery food was related to the prevalence of depression. A study of a Spanish cohort reported that the consumption of fast foods and processed pastries increased the risk of depression, which could be caused by high-fat consumption and enhanced cardiovascular risk^30^.

Alcohol consumption can increase the probability of depression development^31,32^; after drinking alcohol, the subsequently reduced serotonin levels in the blood could cause depression^33^. Serotonin deficiency is the main pathophysiology of depression^34^. People who consume large amounts of alcohol present different serotonin levels in their brains compared to those who consume less or no alcohol. Further, there exists a bidirectional relationship between alcohol consumption and depression, namely each can cause the other^35^.

Generally, social gathering and active contact with neighbors have positive effects on depression^36,37^. However, different from previous results, in our study depression was less frequent among individuals who had decreased contact with neighbors and friends due to the COVID-19 pandemic. A recent commentary on depression during COVID-19 proposed that the prevalence of depression might not have been high in early surveys of the COVID-19 pandemic because people might have adapted to the negative life changes that occurred in response to the pandemic^38^. Furthermore, our result might have been due to reverse causality because it was a cross-sectional survey. Since we could not evaluate the individuals’ social contact before the COVID-19 pandemic, it is possible that people having rare social contact due to depressive mood answered that their contact with neighbors was unchanged due to COVID-19.

Our study was conducted based on a nationwide survey of the Korean population, which revealed an association between depression and daily life restrictions due to the COVID-19 pandemic. There are a few limitations to our study. First, since this study was a cross-sectional study, we could not evaluate the longitudinal effect of daily life restrictions between pre- and post-COVID-19. Thus, the clear causality of increasing depression due to COVID-19 and daily life restrictions remains to be shown in further studies. Second, we measured daily life restrictions subjectively using a structured questionnaire, which might not have fully reflected individuals’ perspectives. Since the definition of depression was assessed using the Korean version of the PHQ-9, the evaluation might have differed slightly from the DSM-V criteria for depression. Finally, we assumed that the cause of daily life restrictions was COVID-19 or the social distancing policy in Korea. However, it is challenging to determine which was the main cause of daily life activity constraints and to know which came first.

In conclusion, this study warns of the negative effect of daily life restrictions due to COVID-19 and policies for COVID-19 control. Lifestyle changes such as decreased physical activity increased consumption of delivery food, and increased fast food consumption also increased OR for depression. Therefore, it is necessary to consider the mental health of vulnerable groups that are particularly affected by the COVID-19 pandemic and the policies enacted for its control. In addition, it is important to maintain lifestyle factors helpful for depression.

## Data Availability

The datasets generated during and/or analysed during the current study are available in the Korean Health Community Survey repository and can be downladed from https://chs.kdca.go.kr/chs/main.do (available in Korean). The English version of the datasets generated during and/or analyzed during the current study are available from the corresponding author on reasonable request.
